# Salmagundi

**Published:** 1888-07

**Authors:** 


### SALMAGUNDI.

EDITED BY E. G. BETTY, D.D.S.

JUNE 2nd, was a happy day in the family of Dr. Heise, the occasion being the birth of a daughter.

DR. GEO. H. CUSHING, of Chicago, has returned from a trip to the South much improved in health.

THE hot weather is upon us making work irksome--so go to the society meetings--get a rest and learn something new wherewith to tickle the confiding patient.

DR. MATT STOUTE, of Chicago, spent a week in Cincinnati, last month very much to his enjoyment. He talked college nearly all the time--you know they need dental colleges in the Windy City.

WHAT'S this we hear about a new International Dental and Oral Journal? The more the merrier, but it seems to us, that some one ought to devise a scheme for making dentists subscribe liberally for those already established.

PROF. W. D. HALLIBURTON recommends (*Quarterly Journal of Microscopical Science*) the following as an easy way to get Methæmoglobin crystals: Defibrinate a few cubic centimeters of the blood of a rat, guinea-pig or squirrel, and add to it a few drops of amyl nitrite, and shake violently for a minute or two, or until the nitrite assumes a chocolate color. A drop of this is withdrawn, with a pipette and placed on a slide, the cover-glass being applied immediately. In a few moments the methæmogloblin crystals will begin to form. By sealing the edge of the cover-glass, the crystals will remain unchanged a very long time. --*National Druggist.*

SEAMAN, of Howard University, Washington, has secured information from more than twenty American colleges regarding the kind of microscopes used and the results of wear. The answers were, in a large majority, in favor of microscopes of American manufacture.—*Medical News.*

---

THE Ohio State Dental Society will meet in Cincinnati, Oct., 16th, 17th, and 18th. Railroad fares will be very cheap owing to the Centennial Exposition which will hold over until Oct. 27th, thus giving opportunity to dentists from neighboring States to attend. The official call will be issued in due form ere long. Let every dentist in Ohio, Pennsylania, Virginia, West Virginia, Kentucky, Indiana, Illinois, Michigan, Tennessee and other States, make up his mind to attend.

---

HABITUAL constipation may be relieved by injecting into the rectum, by means of an ordinary glass syringe, about one half or a tea spoonful of glycerine. According to Anacker, the explanation is this: Glycerine, when brought into contact with the mucous membrane of the rectum, withdraws water from it, thus causing hyperemia and irritation of the sentient nerves of the rectum, which it in turn leads reflexly to powerful peristaltic contractions, ending in defecation. The larger the accumulation of feces, the greater is the effect. There is no discomfort or pain, but the action takes place quickly, safely, and pleasantly. Sometimes a little throbbing is felt in the rectum for a few minutes afterwards. I feel sure that this plan, on account of its simplicity and readiness, will be found to constitute a veritable improvement in the therapeutics of constipation.

---

ANTIPYRIN AS A SUBSTITUTE EOR MORPHINE.—M. See affirms that antipyrin may with advantage replace morphine in nearly, if not all, cases where that alkaloid is injected subcutaneously for the relief of pain, antyprin being free from many of the objectionable consequences attending the use of the opium alkaloid, especially that known as morphinism. He mentions, among others, a series of cases of articular rheumatism cured by two or three injections of 0.5 gram of antipyrin dissolved in an equal weight of water, aided by the administration of the medicine internally; also the relief of pain in acute and chronic gout, neuralgia, lumbago, angina, etc. The subcutaneous injection of antipyrin is said not to be followed by sleepiness, nor by vomiting or excitement, but after a short sensation of tension there is a considerable remission of pain from whatever cause.—*London Pharm. Jour.*—*Jour. Materia Medica.*

---

How the iodoform acts is not clear. Behring claimed that it was decomposed and set free acetylen; the author failed to distinguish free iodine; he suggests that it may have acted mechanically in the experiments. He summarizes, that, "looked at from the clinical side, the ultimate object of all medical research, the following rules may be accepted:

"1. Iodoform, not being a germicide, is not a fit substance to use to procure asepsis of instruments, materials or wounds.

"2. Iodoform is allowable, with the present state of our pharmacopœia, in infected wounds, where true germicides are contra-indicated, as by danger of poisoning or impracticability.

"3. As has long been known, iodoform has a decided tendency to stop serous oozing, and, therefore, may be indicated in wounds where the moisture threatens the integrity of the aseptic or anticeptic dressing."

Some experiments by the author with iodol and salol gave results similar to those with iodoform. H. M. S.

*American Journal Medical Sciences, January,* 1888.

We still maintain, notwithstanding the above conclusions, that for filling roots of dead teeth, iodoform has no equal; its prevention of serous oozing is a decided advantage.

